# miR-380-3p regulates melanogenesis by targeting SOX6 in melanocytes from alpacas (*Vicugna pacos*)

**DOI:** 10.1186/s12864-019-6343-4

**Published:** 2019-12-10

**Authors:** Xuexian Liu, Bin Du, Pengqian Zhang, Junzhen Zhang, Zhiwei Zhu, Bo Liu, Ruiwen Fan

**Affiliations:** 1 College of Animal Science and Veterinary Medicine, Shanxi Agricultural University, Mingxian South Road, Taigu, China; 2 Department of Ecology Research, Beijing Milu Ecological Research Center, Nanhaizi, Daxing district, Beijing, China; 3 College of Life Science, Shanxi Agricultural University, Taigu, China

**Keywords:** microRNA-380-3p, Sex-determining region Y-box 6, Melanogenesis, Microphthalmia-associated transcription factor, Tyrosinase, Tyrosine-related protein-1, Dopachrome tautomerase

## Abstract

**Background:**

Melanocytes are derived from neural crest stem cells in the embryonic stage. In mature melanocytes, a series of complex enzyme-catalyzed reactions leads to the production of melanins, which determine the hair and skin colors of animals. The process of melanogenesis is complex and can be regulated by mRNA, microRNAs (miRNAs) and long noncoding RNAs (lncRNAs) genes. MiRNAs are a type of endogenous noncoding RNA approximately 22 nt in size that predominantly regulate gene expression by inhibiting translation. miR-380-3p is a candidate miRNA potentially related to melanogenesis. To better understand the mechanism of miR-380-3p melanogenesis regulation, plasmids to overexpress or knockdown miR-380-3p were transfected into alpaca melanocytes, and their effects on melanogenesis were evaluated.

**Results:**

In situ hybridization identified a positive miR-380-3p signal in alpaca melanocyte cytoplasm. Luciferase activity assays confirmed that SOX6 is targeted by miR-380-3p. miR-380-3p overexpression and knockdown in alpaca melanocytes respectively downregulated and upregulated SOX6 expression at the mRNA and protein levels. Additionally, miR-380-3p overexpression and knockdown, respectively, in alpaca melanocytes decreased and increased the mRNA levels of melanin transfer-related genes, including microphthalmia-associated transcription factor (MITF), tyrosinase (TYR), tyrosine-related protein-1 (TYRP1), and dopachrome tautomerase (DCT). In contrast, miR-380-3p overexpression and knockdown respectively increased and decreased the mRNA levels of β-catenin. Additionally, the effect of miR-380-3p on melanogenesis was assessed by Masson-Fontana melanin staining.

**Conclusions:**

The results demonstrated that miR-380-3p targeted SOX6 to regulate melanogenesis by influencing β-catenin and MITF transcription and translation, which reduced the expression of downstream genes, including TYR, TYRP1, and DCT. These results provide insights into the mechanisms through which miR-380-3p controls melanogenesis.

## Background

Alpaca (*Vicugna pacos*) is a fiber-producing camelid with 22 coats of hair determined by genetic and endocrine factors [1]. An increasing number of genes have been reported to influence mammalian hair color formation. Microphthalmia-associated transcription factor (MITF) is one of these genes and is an important transcription factor that regulates the key enzymes of melanin production, including tyrosinase (TYR), tyrosinase-related protein 1 (TYRP1) and dopachrome tautomerase (DCT) [2]. Moreover, MITF is involved in several pathways that regulate melanogenesis via mRNA, miRNA and lncRNA encoding genes.

miRNAs are essential regulators of cell proliferation and differentiation during development. miRNA biogenesis involves precursor miRNA cleavage into mature miRNAs in a process catalyzed by endonucleases such as Dicer [3]. miRNAs control a wide range of biological processes, including development, differentiation, proliferation and stress response [4]. Numerous miRNAs have been reported to be involved in the regulation of melanogenesis [5], and these miRNAs include miR-137, miR-434-5p, miR-218, miR-21a-5p, miR-145, miR-340 and miR-27a-3p [6–12]. We have demonstrated that miR-508-3p [13] and miR-25 [14] regulate melanogenesis by targeting MITF in alpaca melanocytes. miR-143-5p regulates alpaca melanocyte migration, proliferation, and melanogenesis [15]; miR-5110 regulates eumelanin production and transfer in alpaca (*Vicugna pacos*) melanocytes by cotargeting melanophilin (MLPH) and Wnt family member 1 (Wnt1) [16]; and lpa-miR-nov-66 targeting of soluble guanylate cyclase (sGC) regulates melanogenesis via the cyclic adenosine monophosphate (cAMP) pathway [17]. To identify more miRNAs that regulate melanogenesis, we identified a potential melanogenesis regulator, miR-380-3p, by comparing the miRNA expression profiles of sheep skins with different hair colors in a previous experiment (unpublished data). miR-380-3p, also known as miR-380 [18], has been found to be expressed in the brain of mice [19] and is differentially expressed in the medial nasal processes in the fetal tissue between gestational days 10 and 11.5 [20]. This miRNA is not affected by Dicer ablation in Sertoli cells [21], suggesting that it is generated by Dicer-independent miRNA biogenesis pathways, such as the Ago2 pathway [22]. We used a bioinformatic analysis to predict if miR-380-3p may targets the sex-determining region Y-box 6 (SOX6) gene. SOX6 and members of the SOXD group, such as SOX5 and SOX13 [23], act as either positive or negative transcription modulators via various mechanisms [24]. SOX5 and SOX6 are coexpressed with SOXE genes in melanocytes [25]. SOX6 promotes β-catenin degradation by inhibiting the Wnt signaling pathway in adipogenesis [26]. In addition, Wnt/β-catenin signaling is involved in melanogenesis [27]. Therefore, it was deduced that SOX6 may have roles in melanogenesis. We found that SOX6 is expressed and can act as a target for phosphorylation by cyclin-dependent kinase 5 (CDK5) in alpaca melanocytes. Furthermore, SOX6 positively regulates melanogenesis by binding to β-catenin to regulate cyclin D1 [28]. However, CDK5 regulates melanogenesis and changes hair colors in mice [29]. The present study was undertaken to test the hypothesis that miR-380p might play a role in melanogenesis by targeting SOX6 in alpaca (*Vicugna pacos*) melanocytes.

## Results

### miR-380-3p and SOX6 expression in alpaca skin and melanocytes

**Fig. 1:**
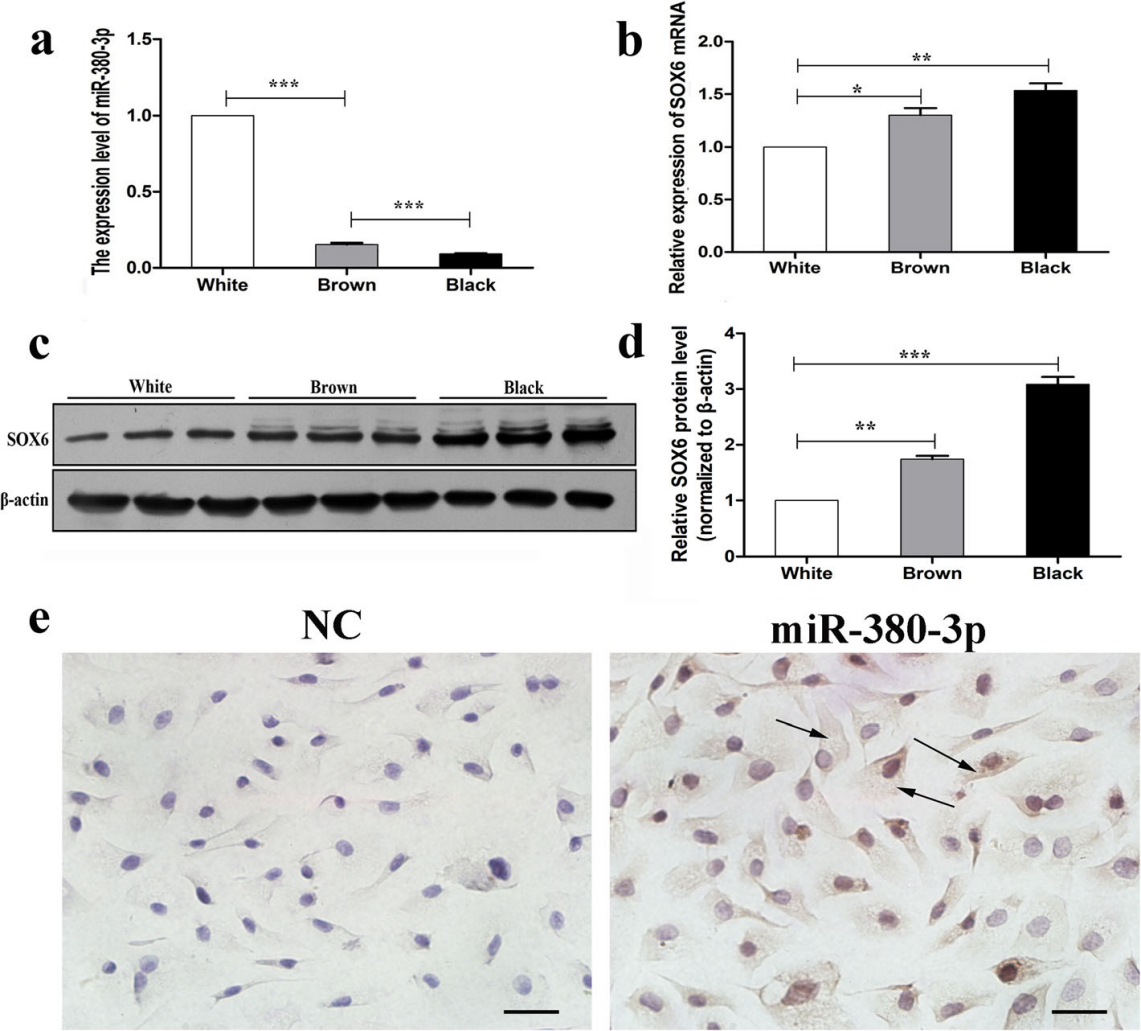
miR-380-3p and SOX6 expression in alpaca skin samples with different hair colors and in melanocytes. **a** RT-qPCR analysis of miR-380-3p expression in alpaca skin samples with different hair colors. **b** RT-qPCR analysis of SOX6 mRNA expression in alpaca skin samples with different hair colors. **c** Western blot analysis of SOX6 protein expression in alpaca skin samples with different hair colors. **d** Quantitative analysis of SOX6 protein expression in alpaca skin samples with different hair colors. **e** Localization of miR-380-3p in alpaca melanocytes in vitro by in situ hybridization analysis. miRNA, mRNA and protein expression levels were normalized to those of U6 mRNA, 18S rRNA and β-actin, respectively. The bars represent the mean ± standard error (*n* = 3). *P < 0.05, **P < 0.01, ***P < 0.001

To confirm the relationship between miR-380-3p and hair color, the expression of miR-380-3p was analyzed by quantitative real-time PCR (RT-qPCR) in alpaca skin samples with white, brown and black hair. The results showed that miR-380-3p expression was significantly higher (*P* < 0.01) in alpaca skin samples with white hair than in the skin samples with black hair and brown hair (Fig. 1a). Additionally, we found that SOX6 levels were lower in alpaca skin samples with white hair than in skin samples with black hair and brown hair at the mRNA and protein levels (Fig. 1b-d, *P* < 0.01). In situ hybridization of miR-380-3p in alpaca melanocytes showed that miR-380-3p is localized in the cytoplasm of these cells, whereas no specific hybridization signal was detected in the cells hybridized with the negative control (NC) probe (Fig. 1e).

### miR-380-3p targets the predicted mRNA binding site in the 3′-UTR of SOX6

**Fig. 2:**
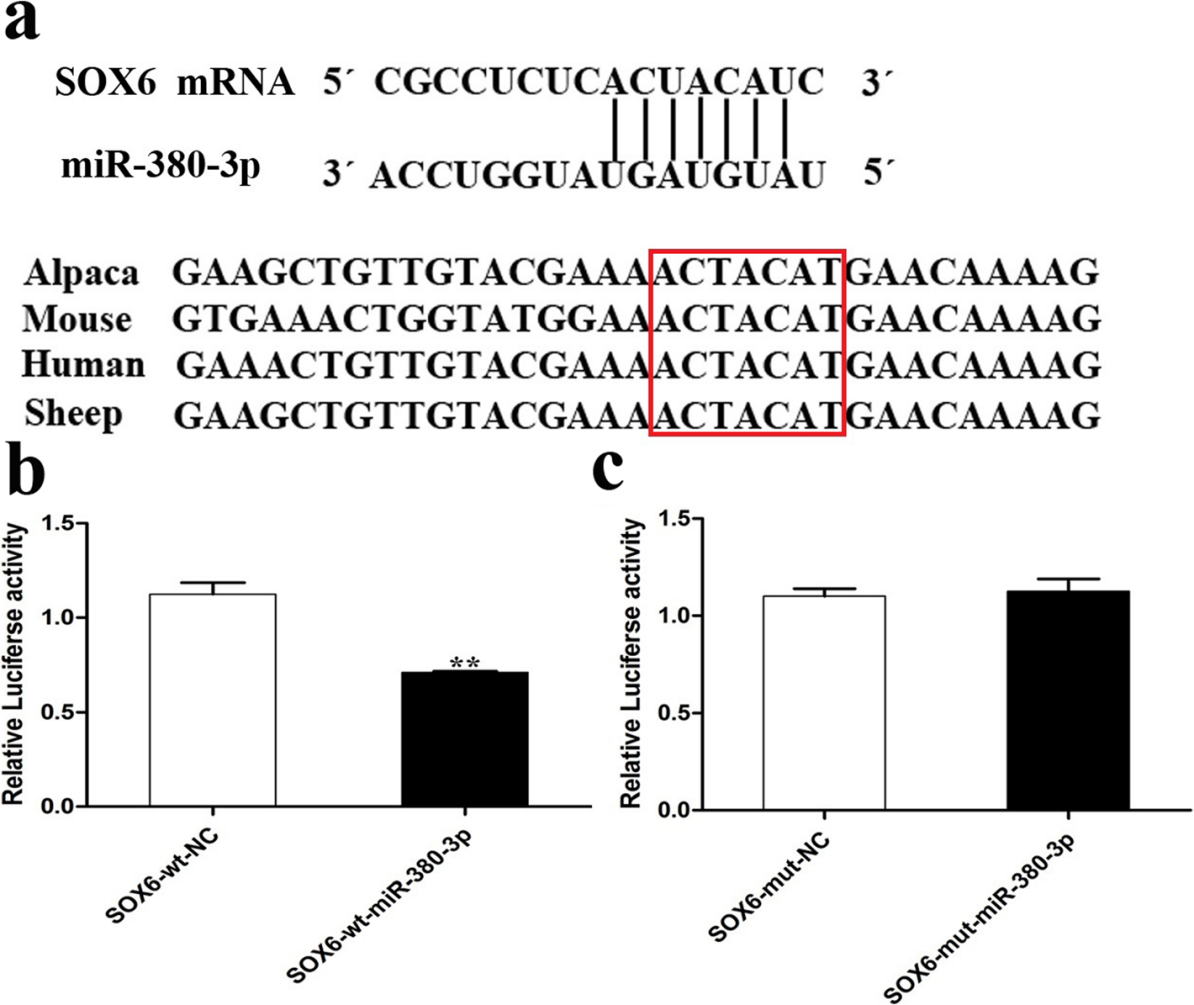
miRNA-380-3p directly targets SOX6. **a** miR-380-3p seed sequence and binding site in the SOX6 3′ UTR of various mammal species. **b**, **c** Luciferase assays in 293T cells cotransfected with miR-380-3p or a negative control (NC) and the luciferase reporter constructs containing either the wild-type SOX6 3′-UTR (SOX6-wt) or a mutated 3′-UTR (SOX6-mut). **P < 0.01

The miRBase (http://www.mirbase.org/), miRTarBase (http://www.mirtarbase.mbc.nctu.edu), and miRDB (http://www.mirdb.org/) databases were used to predict the binding site of miR-380-3p in the SOX6 mRNA. The predicted seed sequence of miR-380-3p is conserved in various mammalian species and is complementary to the predicted binding site in the 3′- untranslated region (UTR) of SOX6 (Fig. 2a). To assess whether miR-380-3p regulates SOX6 at the predicted binding site, luciferase reporter assays were performed in 293T cells cotransfected with the miR-380-3p expressing vector and luciferase reporter constructs containing the wild-type SOX6 3′-UTR sequence or mutated binding sites. miR-380-3p overexpression suppressed the reporter signal by approximately 40% (*P* < 0.01) when the wild-type UTR was used, whereas the luciferase activity did not change when the mutant binding sites were used (Fig. 2b). These data indicate that miR-380-3p binds to and regulates SOX6 in a sequence-specific manner through the predicted 3′-UTR binding site.

### Effect of miR-380-3p on SOX6 mRNA and protein expression

**Fig. 3:**
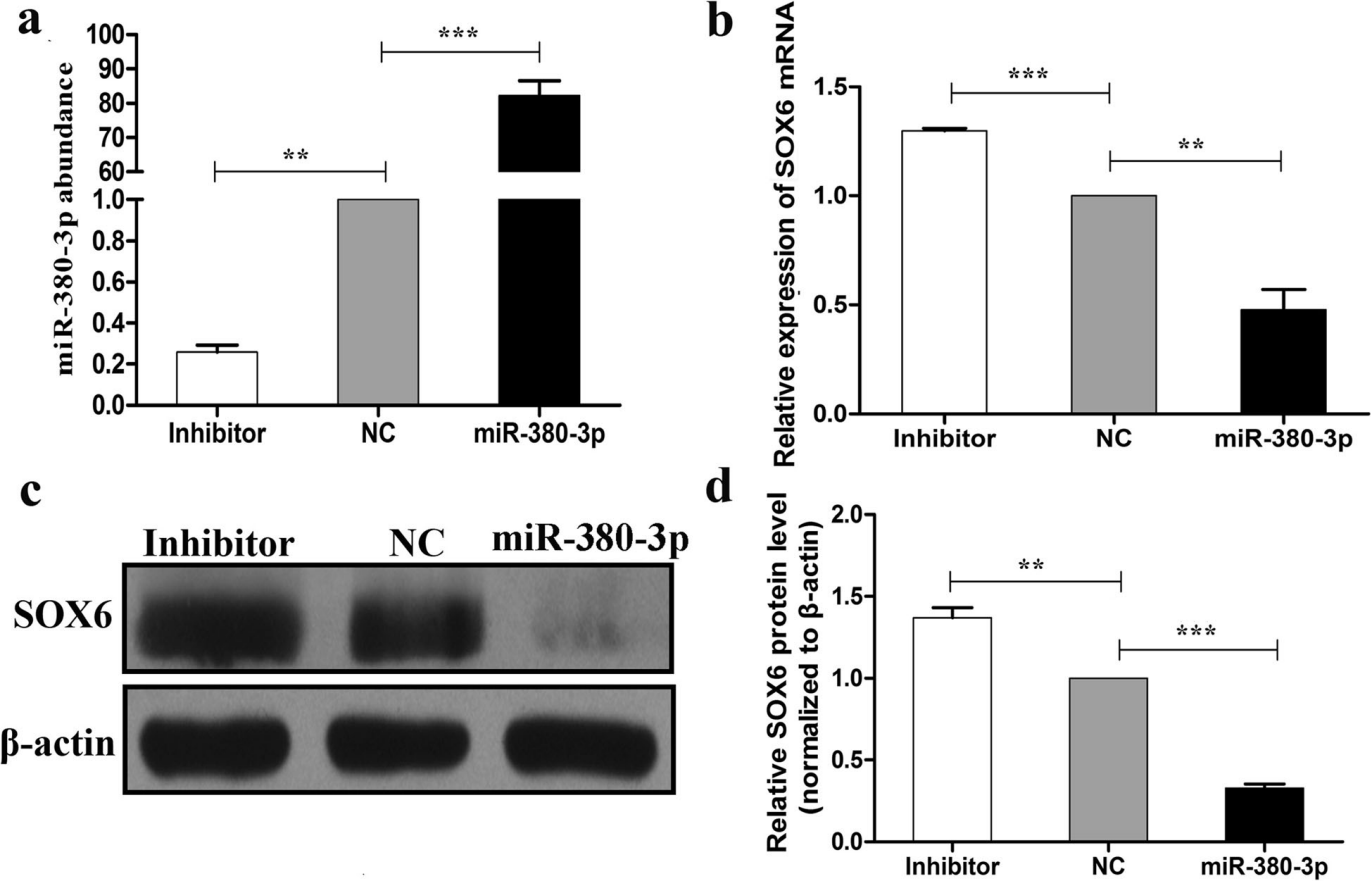
Expression of miR-380-3p and SOX6 in alpaca melanocytes transfected with miR-380-3p. **a** RT-qPCR analysis of miR-380-3p expression in melanocytes transfected with miR-380-3p and its inhibitor. **b** RT-qPCR analysis of SOX6 mRNA expression in melanocytes transfected with miR-380-3p and its inhibitor. **c**, **d** Western blot analysis and quantitative analysis of SOX6 protein expression in melanocytes transfected with miR-380-3p and its inhibitor. mRNA and protein expression levels were normalized to those of 18S rRNA and β-actin, respectively. The bars represent the mean ± standard error (*n* = 3). *P < 0.05, **P < 0.01, ***P < 0.001

To evaluate whether high levels of miR-380-3p and low levels of SOX6 in alpaca skin with white hair are associated, plasmids expressing miR-380-3p or its inhibitor were transfected into alpaca melanocytes, and SOX6 expression was evaluated. RT-qPCR data indicate a significant increase in miR-380-3p expression in melanocytes transfected with the miRNA expression plasmid compared to that in the cells transfected with the NC plasmid (Fig. 3a, *P* < 0.001). miR-380-3p overexpression resulted in a significant decrease in SOX6 abundance at the mRNA (Fig. 3b, *P* < 0.01) and protein levels (Fig. 3c-d, *P* < 0.01). These data suggest that miR-380-3p targets SOX6.

### Effect of miR-380-3p on the expression of β-catenin and melanogenic genes

**Fig. 4:**
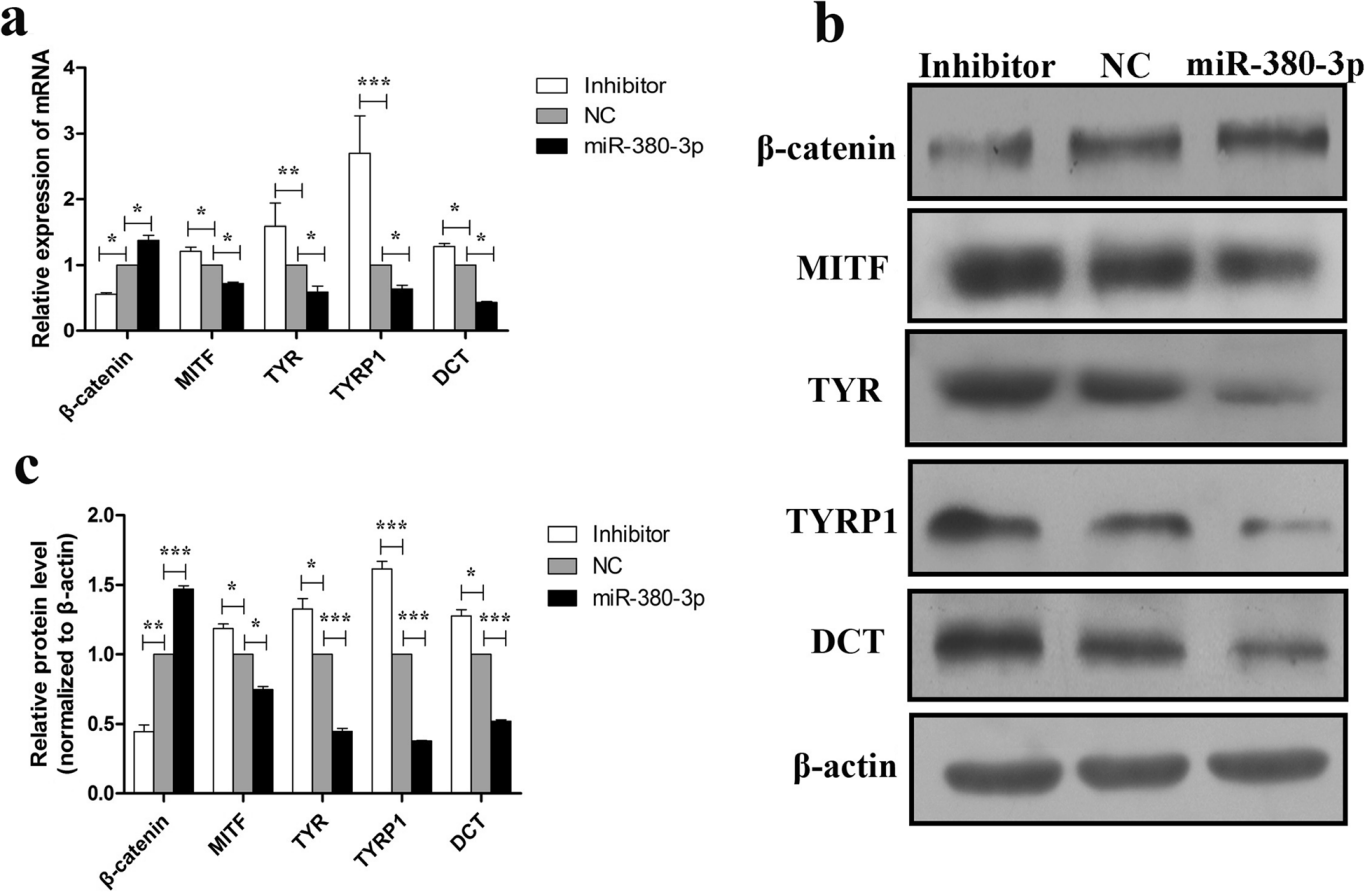
Effect of miR-380-3p on mRNA and protein expression levels of β-catenin and melanogenic genes. **a** RT-qPCR analysis of β-catenin, MITF, TYR, TYRP1, and DCT mRNA expression levels in melanocytes transfected with miR-380-3p and its inhibitor compared with those in negative control (NC) melanocytes. **b**, **c** Western blot and quantitative analysis of β-catenin, MITF, TYR, TYRP1, and DCT protein levels in melanocytes transfected with miR-380-3p and its inhibitor compared with those in NC melanocytes. mRNA and protein expression levels were normalized to 18S rRNA and β-actin expression, respectively. The bars represent the mean ± standard error (*n* = 3). *P < 0.05, **P < 0.01, ***P < 0.001

SOX6 has been shown to interact physically with β-catenin in pancreatic β-cells [30]. To determine whether SOX6 has similar effects on β-catenin in melanocytes, β-catenin mRNA and protein levels were examined in melanocytes transfected with miR-380-3p and inhibitor plasmids. The results showed that β-catenin expression is significantly upregulated or downregulated at the mRNA and protein levels by miR-380-3p overexpression or knockdown vectors, respectively. Additionally, the expression levels of typical melanogenic genes, including MITF, TYR, TYRP1, and DCT, were significantly decreased or increased in melanocytes transfected with miR-380-3p overexpression or knockdown vectors, respectively, compared with those in cells transfected with the NC plasmid (Fig. 4a-c, *P < 0.05, ** *P* < 0.01, *** *P* < 0.001).

### Effect of miR-380-3p on melanin production

**Fig. 5:**
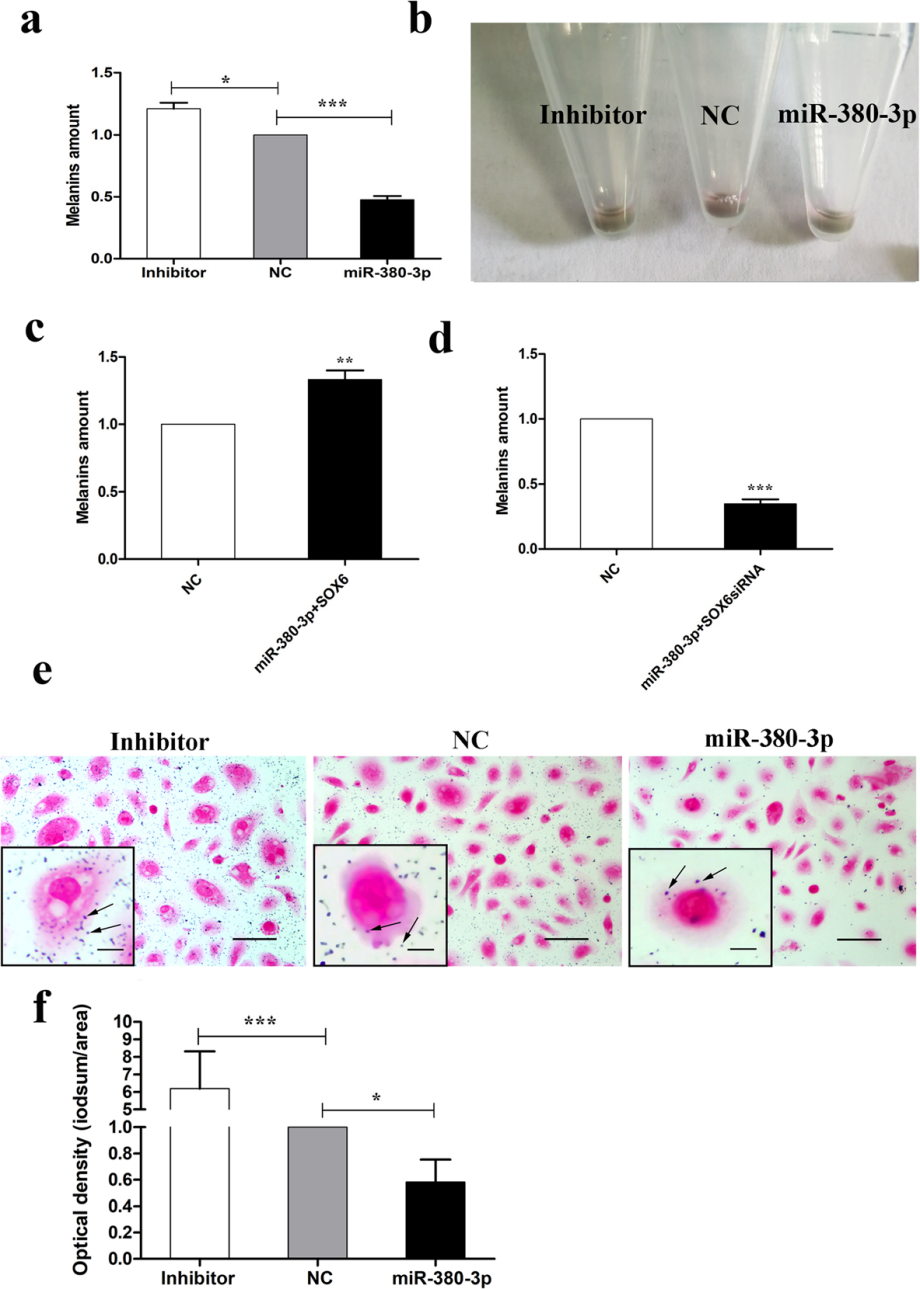
Effect of miR-380-3p on melanin production in alpaca melanocytes. **a** Effect of miR-380-3p overexpression and knockdown on melanogenesis compared with negative control (NC) conditions. **b** Effect of miR-380-3p on cell pellets. **c** Effect of miR-380-3p overexpression and SOX6 on melanogenesis compared with negative control (NC) conditions. **d** Effect of miR-380-3p overexpression and SOX6 siRNA on melanogenesis compared with negative control (NC) conditions. **e** Effect of miR-380-3p overexpression and inhibition in melanocytes on melanin according to Fontana-Masson staining. **f** Quantitative analysis of the distribution of melanin particles. The bars represent the mean ± standard error (*n* = 3). ***P < 0.001

To determine whether miR-380-3p influences melanin production, total alkali-soluble melanin was quantified in alpaca melanocytes transfected with the miR-380-3p expression plasmid and its inhibitor. miR-380-3p overexpression and knockdown in alpaca melanocytes reduced alkali-soluble melanin production by 50% and increased alkali-soluble melanin production by 1.25-fold over what was observed in the control group (Fig. 5a, *P* < 0.001). Next, melanocytes were collected by centrifugation (Fig. 5b). To verify the effect of miR-380-3p, we performed miR-380-3p and SOX6 cotransfection in the melanocytes. The results showed that miR-380-3p and SOX6 can rescue melanin production (Fig. 5c-d, *P* < 0.001). Masson-Fontana melanin staining indicated that the number of melanin particles (arrow) was significantly decreased after miR-380-3p overexpression (Fig. 5e, f, *P* < 0.001).

## Discussion

Melanocytes are melanin-producing cells responsible for skin and hair pigmentation. They contribute to the appearance of the skin and provide protection from ultraviolet radiation damage [31]. Numerous studies have focused on identifying the genes that regulate melanogenesis because of their involvement in melanoma formation. A number of precise pigmentation mechanisms determine the color of the skin or hair of alpacas, which have 22 natural hair colors; microRNAs play important roles in these effects. miR-380-3p is not expressed in normal human melanocytes, but it is expressed in melanoma cell lines [32]. However, our results indicate that miR-380-3p is expressed in normal alpaca melanocytes and is localized in the cytoplasm, suggesting that miR-380-3p plays a certain role in melanocyte biology in alpacas.

Using bioinformatics tools, we predicted that SOX6 is a target gene of miR-380-3p, suggesting that the interaction between miR-380-3p and SOX6 is conserved in mammals. Additionally, we found that SOX6 is differentially expressed at the mRNA and protein levels in alpaca skin with different hair colors. miRNAs in animals are considered to repress target mRNA expression at the translation level with little or no decrease in mRNA abundance; however, an increasing number of studies have revealed that mRNA destabilization can be promoted by miRNAs via the GW182 protein (TNRC6A–C in mammals and GW182 or Gawky in Drosophila) [33]. This relationship suggests that miR-380-3p can not only repress SOX6 translation but also induce SOX6 mRNA decay. SOX6 is a novel gene regulating melanogenesis, as discovered in our previous study [28]. We have found that SOX6 downregulation leads to an increase in β-catenin expression and is accompanied by a decrease in cyclin D1 expression in melanocytes [28]. Thus, the effect of miR-380-3p on β-catenin expression is apparently mediated by SOX6 via the cyclin D1/β-catenin pathway. Previous studies have shown that the MITF promoter is potently activated by β-catenin in B16 melanoma cells and melanocytes [34, 35]. It has also been reported that in numerous types of cancer, including melanoma, decreased MITF expression is induced by the Wnt/β-catenin pathway [36].

**Fig. 6:**
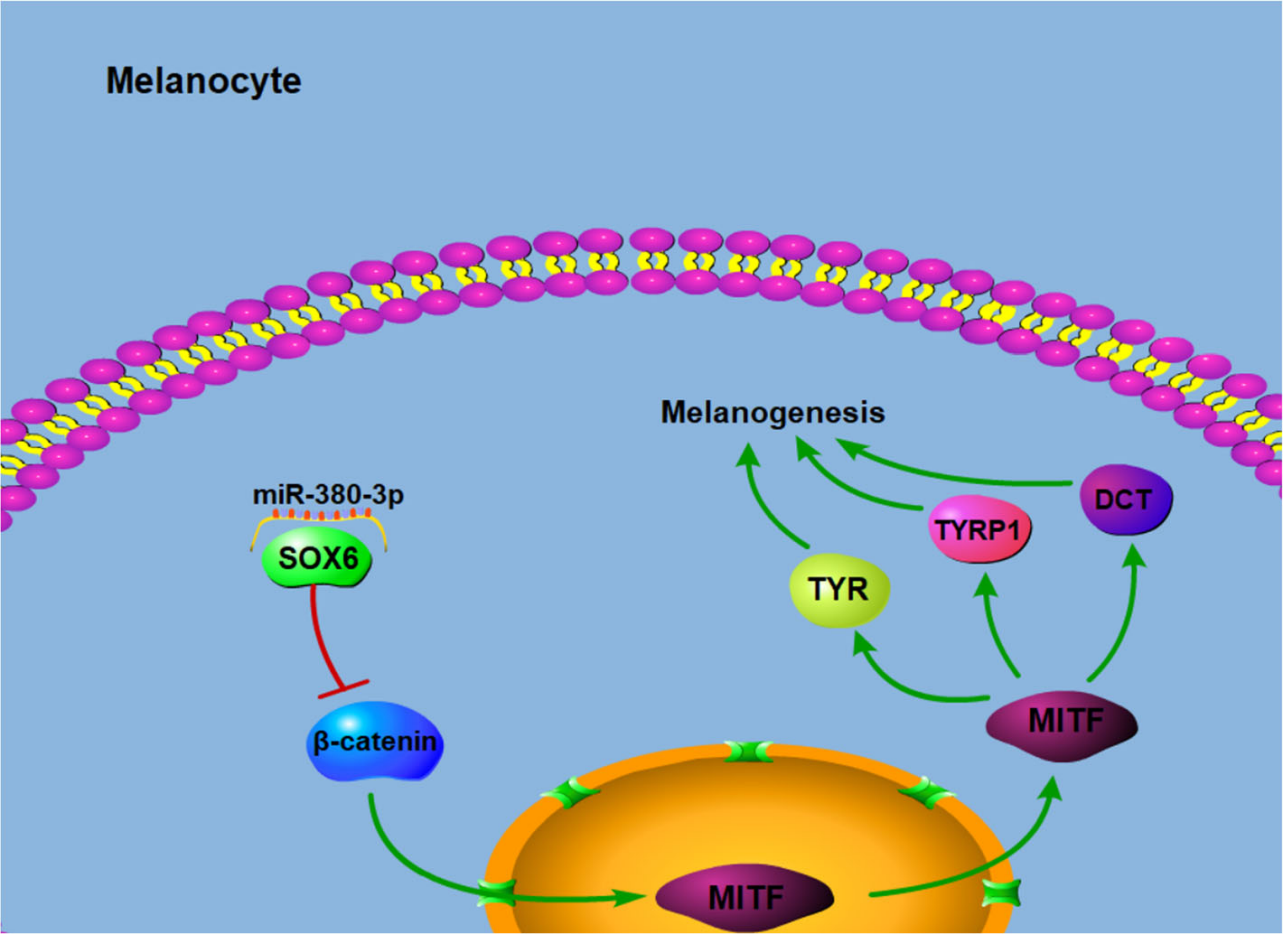
Schematic representation of a potential pathway through which miR-380-3p may regulate melanogenesis based on the results of this study

Activation of the Wnt/β-catenin pathway stabilizes β-catenin in the cytoplasm, leading to its translocation into the nucleus where it associates with TCF/LEF transcription factors and activates the expression of MITF gene [37]. All these studies point to a positive relationship between the expression of β-catenin and MITF. However, our data showed that upregulation of β-catenin was associated with decreased expression of MITF. We cannot provide a good explanation for this result. However, it is possible that SOX6 may regulate the expression of MITF directly. Indeed, MITF is a direct target gene of SOX10, a member of the SOXD family, which is essential for melanocyte development [38]. MITF is known to regulate the transcription of three genes encoding major pigmentation enzymes: TYR, TYRP1, and DCT [39]. Consequently, MITF downregulation in alpaca melanocytes during miR-380-3p overexpression results in a decrease in TYR, TYRP1, and DCT expression at the mRNA and protein levels, which in turn results in reduced melanin production (Fig. 6).

## Conclusions

Our results demonstrate that miR-380-3p binds to the 3’UTR of SOX6 mRNA, thus downregulating its expression at the mRNA and protein levels. The downregulation of SOX6, in turn, affects β-catenin and MITF transcription and translation, causing a reduction in melanin production mediated by the downstream genes TYR, TYRP1, and DCT. These results indicate that miR-380-3p plays an important role in melanogenesis and provide insight into the mechanisms through which miR-380-3p controls melanogenesis by targeting SOX6.

## Methods

### In situ hybridization

Punch skin biopsies (4 × 8 mm) were obtained from three healthy male 1-year-old alpacas with white, brown (spectrophotometric assay for alkali-soluble melanins 1.27~1.95 and eumelanin 0.47~0.78) and black hair colors from an alpaca farm of Shanxi Agricultural University. The biopsies were obtained after local anesthesia by subsequent injection of 2 ml of xylazine hydrochloride (0.05 mg/ml) into the cervical muscles of the alpacas. After 15 min, 1 ml of a normal saline solution containing 800,000 units of penicillin and 400,000 units of streptomycin was mixed with 1 ml of 2% lidocaine (20 mg/ml) at a ratio of 1:1, and 0.5 ml of the mixed solution was then injected at each of three skin sampling sites. The samples were immersed immediately in melanocyte basal medium (Promocell GMBH, Heidelberg, Germany) containing 25 mM N-(2-hydroxyethyl)-piperazine-N′-(2-ethanesulfonic acid) (HEPES), 400 U/ml penicillin and 400 μg/ml streptomycin and transported to the laboratory on ice. The alpaca melanocytes were prepared as previously described [40]. miR-380-3p expression in melanocytes was evaluated by in situ hybridization. In detail, melanocytes were treated with proteinase K (Roche Applied Science, Shanghai, China), washed three times with 0.1 M phosphate-buffered saline (PBS) (Solarbio, Beijing, China) and fixed in 4% paraformaldehyde for 30 min on slides. After the cells were prehybridized with 20 μl of the prehybridization buffer for 3 h, 3 pmol of a digoxigenin-labeled miR-380-3p probe was added to 20 μl of the hybridization buffer to carry out the hybridization at 38 °C overnight. The slides were washed twice with SSC at 37 °C and incubated with 1:1000 diluted alkaline phosphatase-conjugated mouse anti-digoxigenin antibody (Roche Applied Science) for 4 h at 37 °C. Finally, diaminobenzidine (DAB) was used as the substrate for the alkaline phosphatase reaction. The miR-380-3p probe sequence was 5′-GTGGACCATACTACATACGACACAGAAG-3′; the NC (scrambled sequence) was 5′-GTGTAACACGTCTATACGCCCA-3′ (Bio-High Technology, Hebei City, China).

### Construction of plasmids

The plasmid containing miR-380-3p was constructed by inserting pre-miR-380-3p oligonucleotides into the pcDNA6.2-GW/EmGFPmiR vector (Invitrogen, Carlsbad, CA, USA). The inhibitor plasmid was constructed in the same vector by inserting the antisense sequence of miR-380-3p to obtain pcDNA6.2-GW/EmGFPmiR380-inhibitor. The NC plasmid was constructed using a scrambled sequence of miR-380-3p.

A luciferase reporter plasmid was constructed by inserting the 3′-untranslated region (UTR) of the alpaca SOX6 gene, including the putative miR-380-3p-binding site, into a dual luciferase pmirGL0 vector (Promega, Madison, WI, USA). Primers containing SacI and XbaI sites were used to amplify the 3′-UTR of the alpaca SOX6 gene (Additional file 1: Table S1) using skin cDNA as a template. The PCR product and the pmirGL0 vector digested with SacI and XbaI were ligated at 22 °C to obtain pmirGL0-SOX6-wt using a T4 ligation kit (Takara, Dalian, China). Additionally, the binding sites of miR-380 targeting the 3′-UTR of SOX6 were mutated using a site-directed gene mutagenesis kit (Beyotime, Shanghai, China), which was ligated with pmirGL0 to construct pmirGL0-SOX6-mut. The alpaca SOX6 coding region was amplified by PCR from cDNA (primers are listed in Additional file 1: Table S1) and cloned into the pcDNA3.1 expression vector with SacI and XbaI restriction sites. SOX6 NC (scrambled) was obtained from Invitrogen (Carlsbad, CA, USA). SOX6-siRNA and NC siRNA were purchased from Sangon Biotech Co Ltd. (Shanghai, China). All constructs were confirmed by sequencing.

### Melanocyte transfection and total RNA and protein preparation

Alpaca melanocytes (10^6^ cells) were cultured in melanocyte medium (MelM) (ScienCell Research Laboratories, Carlsbad, CA, USA) supplemented with 1% melanocyte growth supplement (MelGS), 1% penicillin-streptomycin and 0.5% fetal bovine serum in a 6-well plate in an incubator at 37 °C under an atmosphere of 5% CO_2_.

When the alpaca melanocytes reached approximately 80% confluence, they were transfected with pcDNA6.2-GW/EmGFPmiR-380-3p, pcDNA6.2-GW/EmGFPmiR380-inhibitor, pcDNA3.1-SOX6, SOX6-siRNA or the NC using Lipofectamine 2000 (Invitrogen, Carlsbad, CA, USA) according to the manufacturer’s guidelines. Melanocytes were collected 72 h after transfection, washed 3 times with PBS and divided into two parts for protein and total RNA preparation. For total RNA preparation, 600 μl Trizol (Invitrogen, Carlsbad, CA, USA) was added. After mixing, chloroform was added at a 1:0.2 ratio, mixed vigorously for 30 s, and allowed to stand on ice for 3 min. Then, the samples were centrifuged at 12,000 rpm for 15 min at 4 °C. The aqueous phase was removed, and isopropanol was added at a 1:2 ratio. The samples were then centrifuged at 12,000 rpm for 10 min at 4 °C to obtain pellets; 1 ml of 75% ice-cold ethanol was added to the pellets and centrifuged at 5 °C for 5 min at 4 °C. Finally, 50 μl of diethyl pyrocarbonate (DEPC) water was added to the RNA, which was treated with DNase I (Sigma, St. Louis, MO, USA). For protein preparation, 300 μl of protein lysate and 3 μl of phenylmethylsulfonyl fluoride (PMSF) were added and allowed to stand on ice for 30 min. After centrifugation at 12,000 rpm for 10 min at 4 °C, the supernatants were taken to obtain proteins. The RNA integrity was assessed by gel electrophoresis (Additional file 3: Figure S1), and the concentrations were measured using a NanaDrop 1000 spectrophotometer (NanoDrop, Wilmington, NA, USA) (Additional file 2: Table S2). The protein concentrations were measured by the bicinchoninic acid (BCA) method.

### Dual luciferase assay

For miRNA target validation, 3 μg of pmirGL0-SOX6-wt, pmirGL0-SOX6-mut, pcDNA6.2-miR380-inhibitor and NC were cotransfected into 293T cells using Lipofectamine 2000. Luciferase activities in the transfected cells were measured with a dual luciferase reporter assay kit (Promega) 48 h after cotransfection according to the manufacturer’s instructions. Firefly luciferase activity was normalized to Renilla luciferase activity, and the data are expressed as the relative luciferase activity (mean ± standard error (SE), *n* = 3).

### RT-qPCR assay for miRNA and mRNA expression

For mRNA quantification, 1 μg of total RNA from skin samples from 3 alpacas for each group with white, brown and black hair colors was converted to cDNA using a PrimeScript™ RT reagent kit with gDNA eraser (Takara Code No. RR047A, Dalian, China) according to the manufacturer’s instructions; RT-qPCR was performed using TB Green® Premix Ex Taq™ II (Takara Code No. RR820A, Dalian, China) following the MIQE guidelines. For miR-380-3p quantification, 1 μg of total RNA from skin samples from 3 alpacas for each group with white, brown and black hair colors and melanocytes was converted to cDNA using Mir-X™ miRNA first-strand synthesis and TB Green™ qRT-PCR kits (Clontech Code No. 638313, Dalian, China) according to the manufacturer’s instructions and a specific stem-loop RT primer and common reverse primer according to a previously established method for real-time miRNA quantification [41]. RT-qPCR was then performed using the primer sequences listed in Additional file 1: Table S1 and TB Green® Premix Ex Taq™ II (Takara Code No. RR820A) in a 7500 fast real-time PCR system (Applied Biosystems, Foster City, CA, USA). The thermal profile was 95 °C for 10 min, 95 °C for 30 s, 60 °C for 30 s, and 72 °C for 20 s for 35 cycles. The biological and technical replicates were run in triplicate with melting curves to validate the amplification specificity.

The relative amounts of miRNAs and mRNAs were normalized to the amounts of U6 and 18 s rRNAs, respectively, based on the amplification efficiency test for the target and housekeeping genes. A sample without reverse transcriptase in the reaction was used as a NC. Total RNA from melanocytes transfected with the NC plasmid was used as a calibrator. The miRNA and mRNA transcript abundance was calculated using the comparative threshold cycle (C_T_) method [42] (*n* = 3, mean ± SE). The primer sequences are listed in Additional file 1: Table S1.

### Western blot analysis

Proteins from skin and melanocyte samples were separated with 10% or 8% sodium dodecyl sulfate-polyacrylamide gel by electrophoresis, followed by transfer to polyvinylidene difluoride membranes. The membranes were blocked with 5% skim milk for 2 h and incubated with the following primary antibodies overnight at 4 °C: polyclonal rabbit anti-SOX6 antibody at a 1:1000 dilution (Abcam, Cambridge, MA, USA), polyclonal rabbit anti-β-catenin antibody at a 1:1000 dilution (Abcam), mouse anti-MITF at a 1:1000 dilution (Thermo, Waltham, MA, USA), polyclonal rabbit anti-TYR antibody at a 1:1000 dilution (Abcam), polyclonal rabbit anti-TYRP1 antibody at a 1:1000 dilution (Abcam), and rabbit anti-DCT at a 1:1000 dilution (Abcam). The membranes were washed three times with Tris-buffered saline Tween-20 (TBST) (10 min/wash) and incubated for 1 h at 37 °C with horseradish peroxidase-conjugated secondary antibodies against rabbit or mouse IgG at a 1:10000 dilution (Sigma). The membranes were washed four times with TBST (5 min/wash), and bound antibodies were visualized using enhanced chemiluminescence. Immunoblots were scanned on a ChemiDOC XRS+ imager (Bio-Rad Laboratories, Philadelphia, PA, USA). Protein levels were quantified using Image-Pro Plus software (Olympus, Japan).

### Melanin content measurement

Melanocytes transfected with pcDNA6.2-GW/EmGFPmiR-380-3p, pcDNA6.2-GW/EmGFPmiR380-inhibitor, pcDNA3.1-SOX6, SOX6-siRNA or NC plasmid were harvested and rinsed with PBS, followed by the addition of 1 ml of 1 M NaOH to dissolve melanin. The content of alkali-soluble melanin in the supernatant was measured by a spectrophotometer (Molecular Devices, Sunnyvale, CA, USA) at an absorbance of 475 nm and normalized to the total number of melanocytes. All experiments were performed in triplicate.

### Masson-Fontana staining for melanins

After transfection was terminated, the cells on slides were washed 3 times with PBS for 3 min each time and fixed with 4% paraformaldehyde for 20 min. Then, the slides were treated with a silver ammonia solution and placed in an oven at 60 °C for 45 min. Next, the slides were treated with a gold chloride solution (0.2%) and sodium thiosulphate solution (35.6%) at room temperature for 2 min. A nuclear solid red solution was used for staining, and the slides were sealed with a neutral gum. Three fields of view were selected randomly for each image, and image analysis was performed using Image-Pro Plus software (Olympus, Japan). Intracellular melanin staining was quantified as the integrated optical density (IOD) obtained from the combined area and gray value, and the mean value was calculated.

### Statistical analysis

The data are presented as the mean ± SE. The differences in miRNA, mRNA, and protein contents, relative luciferase activities, and melanin content between the NC and experimental groups were determined by analysis of variance and Fisher’s least significant difference tests using SPSS 17.0 software (Chicago, IL, USA).

## Supplementary information

**Additional file 1: Table S1.** Primers used in the study.

**Additional file 2: Table S2.** Quality of the RNAs.

**Additional file 3: Figure S1.** RNA quality testing.

## Data Availability

All data generated or analyzed during this study are included in this published article and its supplementary information files.

## Abbreviations

BCA: Bicinchoninic acid
cAMP: cyclic adenosine monophosphate
C_T:_: Threshold cycle
DCT: Dopachrome tautomerase
DEPC: Diethyl pyrocarbonate
FSHD: Facioscapulohumeral muscular dystrophy
lncRNA: long noncoding RNA
miRNA: microRNA
MITF: Microphthalmia-associated transcription factor
MLPH: Melanophilin
NC: Negative control
PBS: Phosphate-buffered saline
PMSF: Phenylmethylsulfonyl fluoride
RISC: RNA-induced silencing complex
RT-qPCR: Quantitative real-time PCR
sGC: soluble guanylate cyclase
SOX6: Sex-determining region Y-box 6
TBST: Tris-buffered saline Tween-20
TYR: Tyrosinase
TYRP1: Tyrosine-related protein-1
TYRP2: Tyrosine-related protein-2
UTR: Untranslated region
Wnt1: Wnt family member 1
