# Supplementary material for: miR-380-3p regulates melanogenesis by targeting SOX6 in melanocytes from alpacas (Vicugna pacos)
Source: BMC Genomics. 2019 Dec 10;20:962. doi: 10.1186/s12864-019-6343-4 (PMC6905097; doi:10.1186/s12864-019-6343-4)
Supplement: Supplementary file 1 — Additional file 1: Table S1. Primers used in the study. [file 12864_2019_6343_MOESM1_ESM.docx]

**Table** **S1. Primers used in the study.**

| Primer | Sequence (5′–3′) | Application |
| --- | --- | --- |
| SOX6-F | AAGATGCTGACTGGGACA | Real-time PCR |
| SOX6-R | GGTGAGGTAGAGGTATTTCG | Real-time PCR |
| SOX6-wt-F | CGAGCTCGGACTGTTCTTTAGGACTGA | Luciferase reporter-wt |
| SOX6-wt-R | GCTCTAGATTTAAGAACATGGCTAGGAG | Luciferase reporter-wt |
| SOX6-mut-F | GAAGCTGTTGTACGAAAAGATGATGAACAAAAGTCATCTGT | Luciferase reporter-mut |
| SOX6-mut-R | ACAGATGACTTTTGTTCATCATCTTTTCGTACAACAGCTTC | Luciferase reporter-mut |
| miR-380-3p-RT | CTCAACTGGTGTCGTGGAGTCGGCAATTCAGTTGAG AGACGTGG | Real-time PCR |
| miR-380-3p-F | ACACTCCAGCTGGGTATGTAATGTGGTCC | Real-time PCR |
| Common-R | TGGTGTCGTGGAGTCG | Real-time PCR |
| U6-F | CTCGCTTCGGCAGCACA | Real-time PCR |
| U6-R | AACGCTTCACGAATTTGCGT | Real-time PCR |
| β-catenin-F | GACCCTGCCATCTGTGC | Real-time PCR |
| β-catenin-R | CGGGTGGAGGAGTTTCA | Real-time PCR |
| MITF-F | TCCCAAGTCAAATGATCCAG | Real-time PCR |
| MITF-R | GAGCCTGCATTTCAAGTTCC | Real-time PCR |
| TYR-F | GCTTTAGCAACTTCATGGGA | Real-time PCR |
| TYR-R | CTTGTTCTTCTCTGGGACAC | Real-time PCR |
| TYRP1-F | GCTCAGTGCTTGGAAGTTGGT | Real-time PCR |
| TYRP1-R | AGTTTGTCCTCCAGTTCCGTTTAG | Real-time PCR |
| DCT-F | TGCTTTGCCCTACTGGAAC | Real-time PCR |
| DCT-R | ATCAGAGTCGATCGTCTG | Real-time PCR |
| 18S-F | GAAGGGCACCACCAGGAGT | Real-time PCR |
| 18S-R | CAGACAAATCACTCCACCAA | Real-time PCR |
| SOX6-CDS-F  SOX6-CDS-R  SOX6-siRNA-F  SOX6-siRNA-R | GGGGTACC ATGTCTTCCAAGCAAGCCA  GCTCTAGA CAGTTGGCACTGACAGCT  GCCAACAGCAAGAACAGAUTT  AUCUGUUCUUGCUGUUGGCTT | PCR  PCR  Real-time PCR  Real-time PCR |
