# Supplementary material for: miR-380-3p regulates melanogenesis by targeting SOX6 in melanocytes from alpacas (Vicugna pacos)
Source: BMC Genomics. 2019 Dec 10;20:962. doi: 10.1186/s12864-019-6343-4 (PMC6905097; doi:10.1186/s12864-019-6343-4)
Supplement: Supplementary file 2 — Additional file 2: Table S2. Quality of the RNAs. [file 12864_2019_6343_MOESM2_ESM.docx]

| Sample | Inhibitor | NC | miR-380-3p |
| --- | --- | --- | --- |
| OD_260/280_ | 1.92 | 1.88 | 1.90 |

**Table** **S2. Quality of the RNAs**
