# Supplementary figures and images for: miR-380-3p regulates melanogenesis by targeting SOX6 in melanocytes from alpacas (Vicugna pacos)
Source: BMC Genomics. 2019 Dec 10;20:962. doi: 10.1186/s12864-019-6343-4 (PMC6905097; doi:10.1186/s12864-019-6343-4)

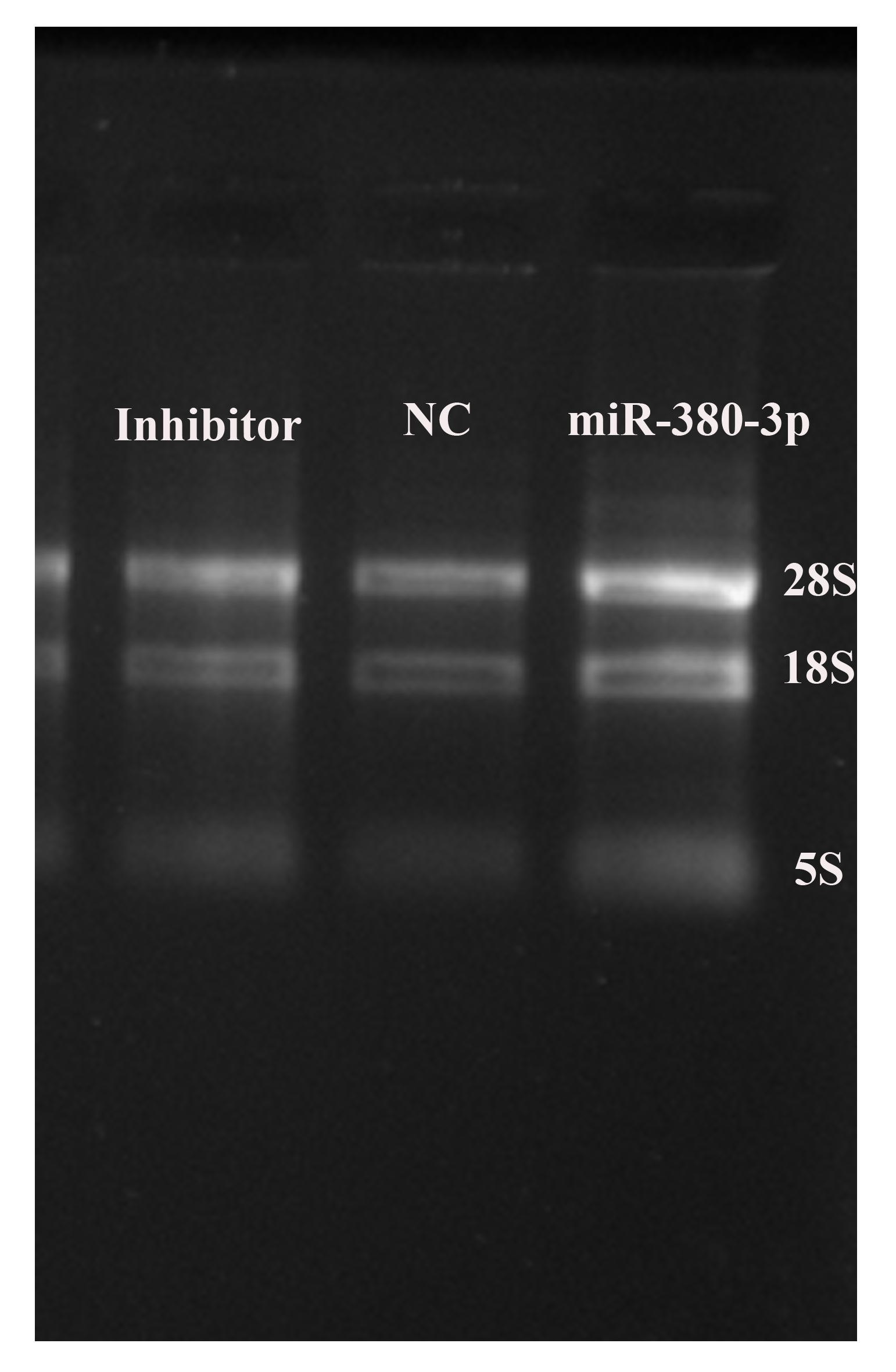

Supplement: Supplementary file 3 — Additional file 3: Figure S1. RNA quality testing. [file 12864_2019_6343_MOESM3_ESM.png]
